# Effectiveness of exercise therapy on pain relief and jaw mobility in patients with pain-related temporomandibular disorders: a systematic review

**DOI:** 10.3389/froh.2023.1170966

**Published:** 2023-07-12

**Authors:** Akiko Shimada, Toru Ogawa, Sara Reda Sammour, Taichi Narihara, Shiori Kinomura, Rie Koide, Noboru Noma, Keiichi Sasaki

**Affiliations:** ^1^ Department of Oral Health Sciences, Faculty of Health Sciences, Osaka Dental University, Osaka, Japan; ^2^ Department of Geriatric Dentistry, Osaka Dental University, Osaka, Japan; ^3^ Division of Advanced Prosthetic Dentistry, Tohoku University Graduate School of Dentistry, Sendai, Japan; ^4^ Prosthodontic Department, Faculty of Dentistry, Tanta University, Tanta, Egypt; ^5^ Department of Oral Medicine, Nihon University School of Dentistry, Tokyo, Japan

**Keywords:** temporomandibular disorder (TMD), myalgia, orofacial pain, exercise theraphy, systematic reveiw

## Abstract

**Background:**

Orofacial pain conditions are complex disorders that involve biological, social, and psychological factors. Temporomandibular Disorders (TMDs) are one of the most common orofacial pain conditions, and our previous literature review indicated that exercise therapy has shown promise in reducing TMD-related pain. However, more evidence is needed to firmly establish its effectiveness.

**Objectives:**

This systematic review aims to investigate the effectiveness of exercise therapy on pain relief and jaw mobility in patients with pain-related TMDs.

**Methods:**

To include randomized controlled trials (RCTs) written in English, a literature search was performed using PubMed, Scopus, Web of Science, Cochrane Library, Ovid, EBM reviews, and Academic Search Complete initially from 4th November 2020 until March 2022. A PICOS for this review was as follows; P: Patients with TMD myalgia or arthralgia, I: Excursion exercise, Stretch exercises, Resistance exercise, or Coordination exercise, C: No treatment or education only. O: Pain intensity and Range of Motion (ROM), S: RCTs. After title screening, a full-text assessment was done to extract data. According to Risk of Bias (RoB) 2.0, risk of bias was assessed in each included paper by 2 reviewers independently.

**Results:**

A total of 3,388 titles were identified from the electronic database search. After the screening and full-text evaluation, only 5 studies (145 participants) were eligible to be included. Among the exercise modalities, coordination exercise showed a significant effect on pain relief and improvement of joint mobility.

**Discussion:**

Due to the heterogeneity and small sample size of the included studies, a meta-analysis was not feasible. However, this systematic review suggested that exercise therapy, especially coordinate exercise, can be effective in managing painful TMD conditions. Further research is needed to establish optimal parameters for this patient population, as well as standardization and consistency in terminology and treatment structure.

## 1. Introduction

Orofacial pain conditions often involve a complex mixture of biological, social, and psychological factors (1). Compared to general dental diseases such as caries or periodontitis, the management or treatment for orofacial pain conditions has not been widely recognized. It is therefore hard for dentists and patients to reach pain-relieving solutions. Even though reversible options should be selected as the first option (2), in some of the worst cases, irreversible treatments such as pulpectomy or tooth extraction are done unnecessarily because of the lack of knowledge on orofacial pain.

One of the most common orofacial pain conditions is Temporomandibular disorders (TMDs) which are characterized by myalgia in the masticatory muscles, and arthralgia in the temporomandibular joints (3). For TMDs, internationally standardized diagnosis criteria, the Diagnostic Criteria for Temporomandibular Disorders (DC/TMD) (4) have been established and widely used both in research and clinical settings. This enabled dentists to make a diagnosis of TMD more accurately regardless of their clinical experience.

Once diagnosed as TMD myalgia or TMD arthralgia, conservative therapy is applied to relieve pain in the masticatory muscles or the temporomandibular joint. The effect of exercise therapy, one of the most common conservative therapy for myalgia and arthralgia in other parts of the body, has also been introduced for TMD (5, 6). According to our previous narrative review (7), among conservative treatment options, exercise therapy demonstrated a possible effect on painful TMDs. The major benefits identified were reduced pain in the masticatory muscles and improvement in the range of movements of the mandible. However, the following concerns were revealed throughout this review process. First, inconsistency in the diagnostic method of TMDs for patients included. Secondly, the modalities of exercises were not standardized. Furthermore, the selection of control groups was quite diverse. Due to such concerns, even though exercise therapy appears to demonstrate effectiveness for TMD myalgia and/or arthralgia, this evidence still needs to be firmly established. Therefore, as a next step, this systematic review was performed, considering such concerns and limiting search conditions more strictly with concrete the Population/Problem, Interventions, Comparison/Control, Outcomes, and Study (PICOS) to eliminate vagueness and unclearness.

This systematic review aims to investigate the effect of exercise therapy on pain relief and improvement of TMJ mobility in patients with painful TMD conditions.

## 2. Methods

This systematic review is reported following the Preferred Reporting Items for Systematic Reviews and Meta-analyses (PRISMA) (8, 9) and performed based on a protocol *a priori* registered on PROSPERO (CRD42020215462).

### 2.1. Study protocol

An electronic search was performed initially on 4th November 2020 up to the period of March 2022 in PubMed, Scopus, Web of Science, Cochrane Library, Ovid, EBM reviews, and Academic Search Complete with the retrieval approach shown in Supplementary Table S1.

This systematic review was conducted based on the following focus questions that are the same as the ones introduced in our previous literature review (7).

• *Is exercise therapy effective to reduce clinical pain intensity in patients with painful TMD compared to the control group investigated by randomized controlled trials (RCTs)?*
• *Is exercise therapy effective to improve jaw movements in patients with painful TMD compared to the control group investigated by RCTs?*

In detail, the following inclusion criteria of the PICOS were decided.

#### 2.1.1. Population/problem

Patients with myalgia or arthralgia, preferably confirmed by the DC/TMD. No age restriction was set.

#### 2.1.2. Interventions

Among modalities of exercise therapy, the following 4 exercise therapies were focused on in this review: excursion exercise for the temporomandibular joint (E1), stretch exercises for the masticatory muscles (E2), resistance exercise for the masticatory muscles (E3), and coordination exercise for jaw open-close movements (E4) (10).

#### 2.1.3. Comparison/controls

No treatment or education only.

#### 2.1.4. Outcomes

Pain intensity.

Range of movements (ROM) of the mandible and/or vertical jaw gape.

#### 2.1.5. Study design

Only RCTs were included in this review.

### 2.2. Inclusion and exclusion criteria

The inclusion criteria for this systematic review were: (1) RCTs and controlled, clinical trials (CCTs); (2) exercise therapy for orofacial pain was described; and (3) written in English.

The exclusion criteria were studies in animals.

### 2.3. Selection criteria

Initially, after duplicates were omitted, studies identified through electronic searches were screened by title and abstract using the following criteria: (1) exercise therapies for orofacial pain were described; and (2) human studies. The screening was performed by two independent reviewers (TO and AS). In case of disagreement, a third reviewer (NN) was consulted to reach a consensus. The reference lists of the included articles were also screened to identify other relevant articles.

### 2.4. Data extraction

Two independent reviewers (SS and RK) extracted the following information from the selected articles, respectively: Study characteristics (first author, publication year, inclusion and exclusion criteria, pre-assessment questionnaires used), participant characteristics (age, sex, TMD conditions), Methods (blinding, randomization, modality of exercise therapy, duration, intensity, targeted area, frequency, and control method), and outcomes (timepoints of assessment and measurement methods of pain intensity and ROM of the mandibular).

### 2.5. Data synthesis

Because PICOS for this systematic review was strictly defined, the heterogeneity in terms of the diversity of exercise modality and time points of assessment was detected. As a result, due to a small sample size, a meta-analysis could not be performed. Therefore, data were synthesized according to the synthesis without meta-analysis (SwiM) (11). All statistical analysis was conducted with Review Manager 5.4.1. Data on pain intensity (Visual Analog Scale: VAS or Numeric Rating Scale: NRS) and ROM (mm) at baseline and the final assessment of an intervention group and a control group in selected studies were compared. Based on the standardized mean difference (SMD) and 95% confidence interval (95% CI) of VAS on pain intensity, as well as ROM, forest plots were provided.

### 2.6. Risk of bias assessment

The risk of bias was assessed by two reviewers (TN and SK) following Risk of Bias 2.0 (RoB 2.0) (12). Both focused parameters, pain intensity, and ROM, were assessed independently. When consensus could not be reached, the disagreement was resolved by discussion between the reviewers.

### 2.7. Assessment of the certainty of the evidence

The GRADE approach was applied to rate the overall certainty of evidence using GRADEpro GDT (https://gradepro.org/) and the guidance in Chapter 14 of the *Cochrane Handbook for Systematic Reviews of Interventions* (Handbook 2021) (13–15). The certainty of evidence reflects the extent to which we are confident that an estimate of effect is correct. For this systematic review, the degree of downgrading was determined by the seriousness of the following factors: (1) risk of bias, (2) inconsistency of results, (3) indirectness, (4) imprecision (insufficient data), and (5) other factors (e.g., reporting bias).

## 3. Results

### 3.1. Study selection

**Figure 1:**
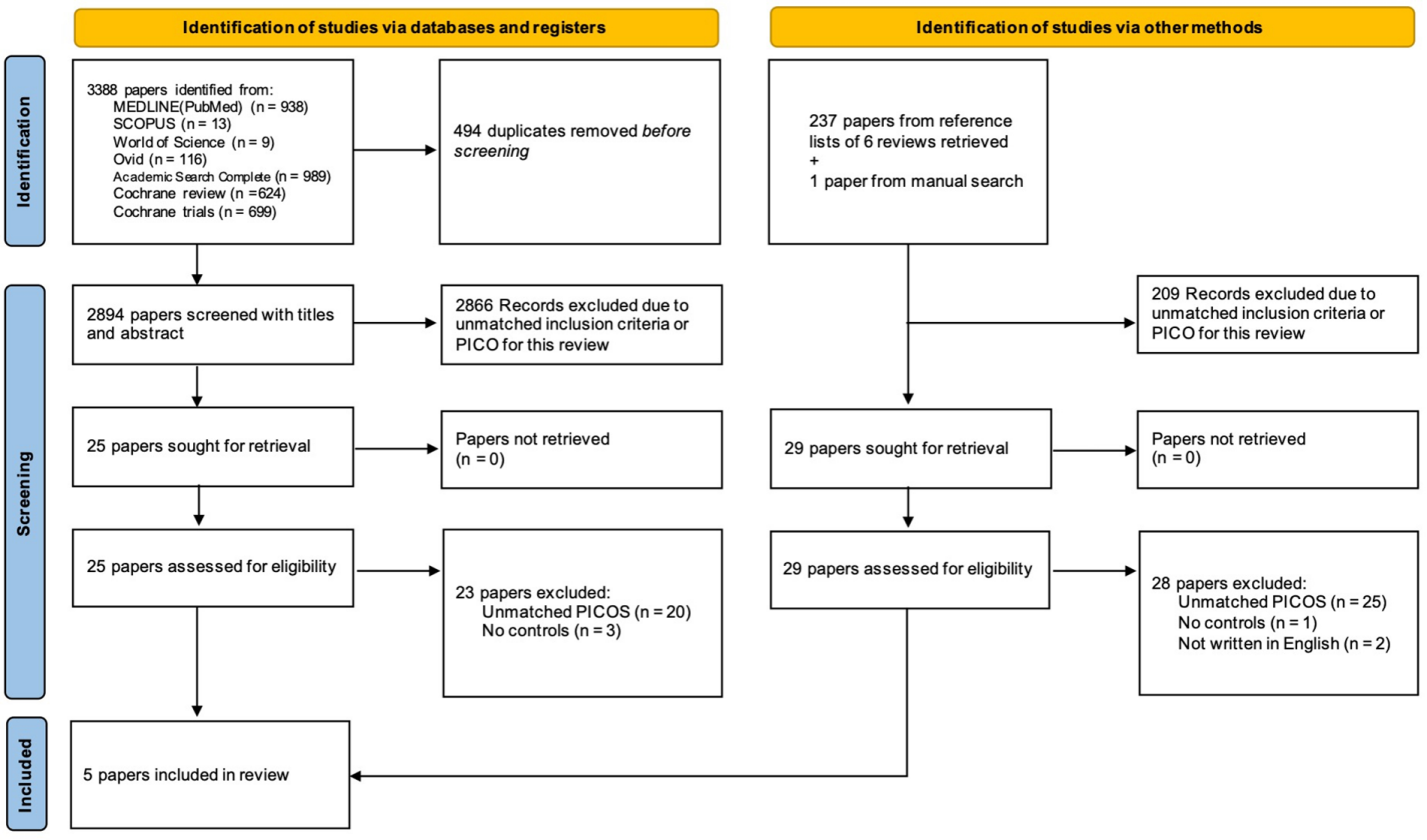
Flow diagram of screening process based on PRISMA guidelines.

Initially, 3,388 titles were identified through electronic searches. After removing 494 duplicates, 2,894 titles were assessed by screening the titles and abstracts. As a result of the title and abstract screening, 28 papers were identified and their full text was obtained for assessment for eligibility for further review performed by 3 independent reviewers (AS, TO, and NN). Finally, out of the 28 papers, a total of 5 papers only were matched the eligibility and included in the qualitative assessment (16–20). The flow diagram of the screening process was shown in Figure 1.

### 3.2. Study characteristics

The characteristics of the eligible studies are summarized in Supplementary Table S2. Participants in 4 studies are patients diagnosed with myalgia disorders (Ia/Ib) according to the Research Diagnostic Criteria for Temporomandibular Disorders (RDC/TMD), whereas one study included patients with limited ROM and pain in TMJ or feeling discomfort with their masticatory function. All modalities of exercise therapy defined as interventions in this review were applied in the selected studies in total. Participants allocated to the control group received either education only or no treatment. The randomized allocation of each group was done in an objective manner such as balanced block randomization or the use of electric randomization methods in all the selected studies, having one exception where it was not explained clearly (19). Regarding the prescription of the introduced exercise program, none of the frequency, intensity, and time were standardized.

### 3.3. Quality assessment

**Figure 2:**
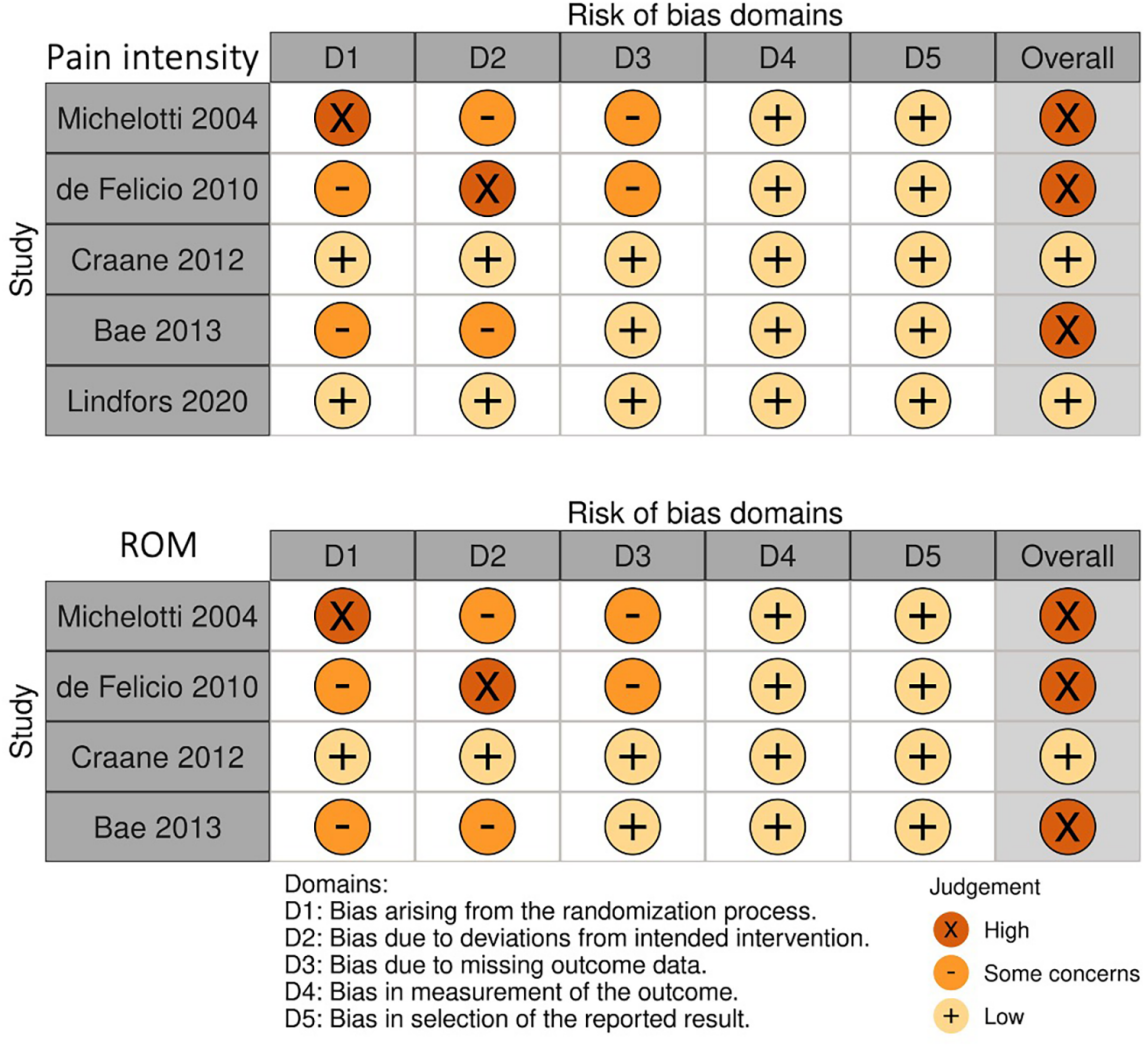
Risk of bias assessment for each included study.

The quality assessment of the included papers was performed based on Risk of Bias 2 (12). Figure 2 shows the risk-of-bias assessment of each paper in all domains. In the overall bias regarding pain intensity assessed in each study, 3 included papers resulted in High risk, whereas 2 papers showed Low risk. In the overall bias regarding ROM, only 1 paper showed Low risk, and the rest of the 3 papers resulted in High risk.

### 3.4. Effectiveness of exercise therapy on pain intensity and range of motion (ROM)

**Figure 3:**
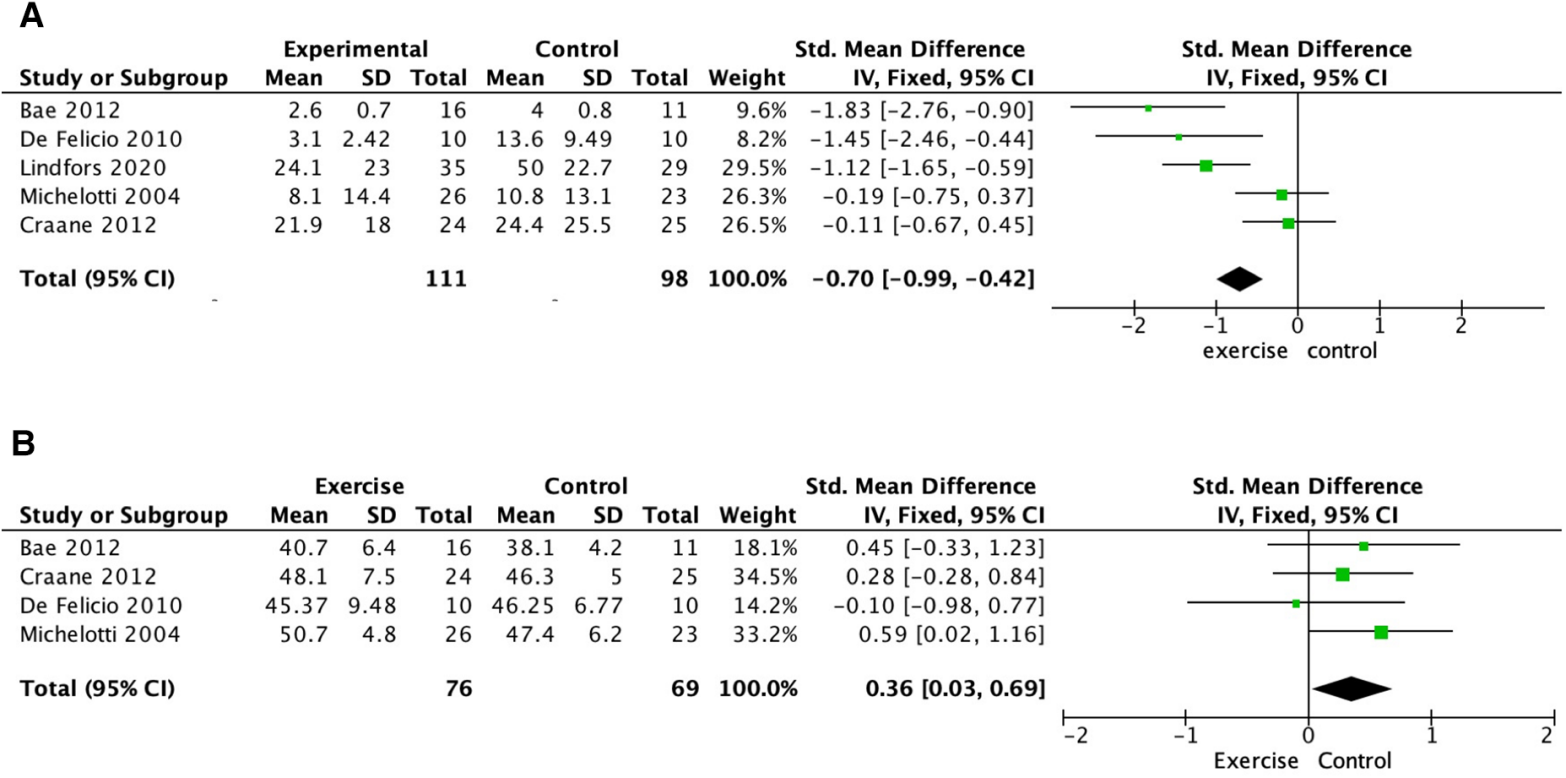
Analysis comparison: exercise therapy compared to no treatment or education. (A) outcome: pain intensity, (B) outcome: ROM.

**Figure 4:**
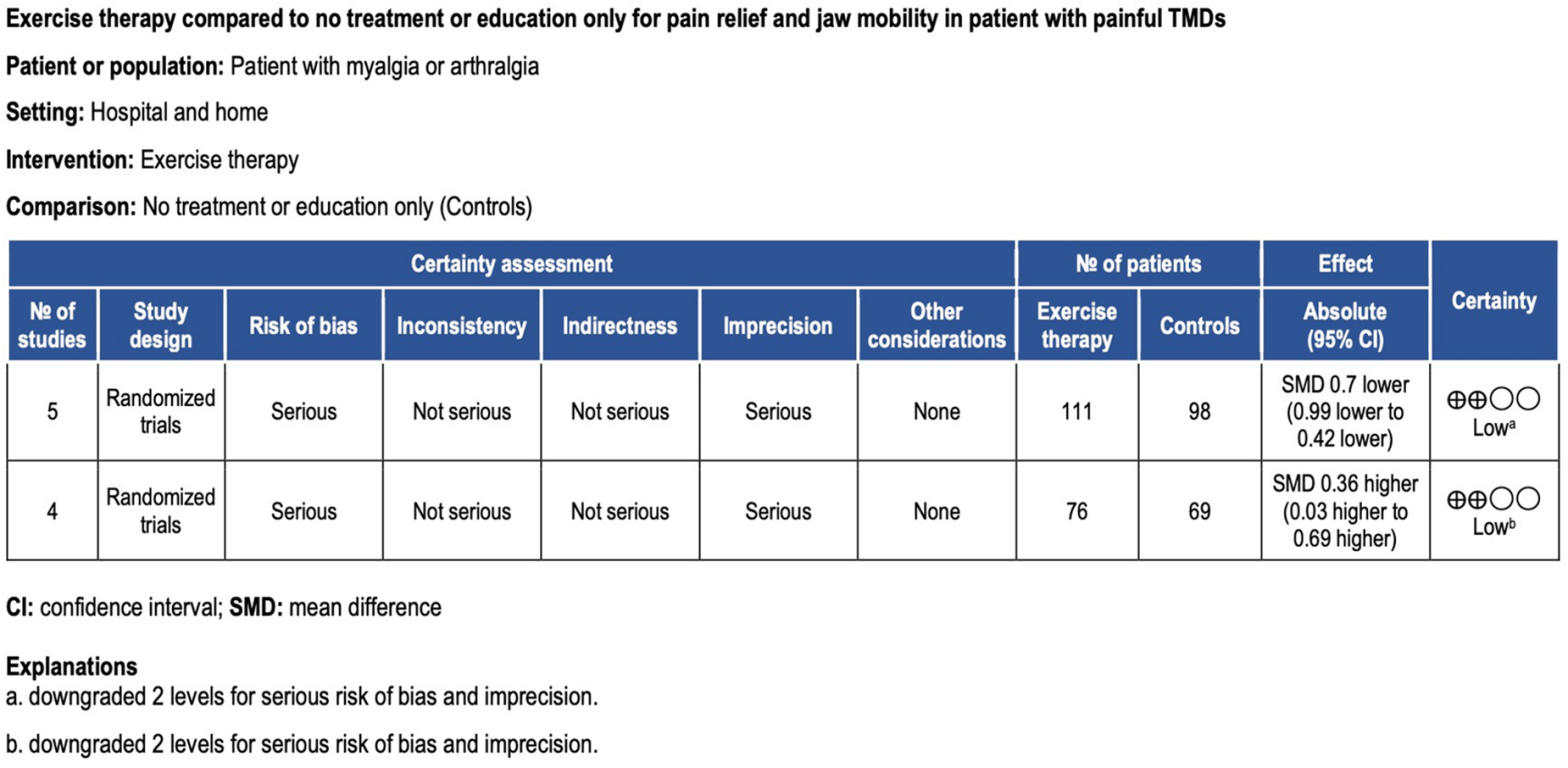
Grade evidence profile.

Pain intensity was assessed by either a 10 mm VAS or a 0–10 NRS in the included studies. In 2 of the included studies where coordination exercise was conducted, pain intensity decreased significantly after exercise therapy (17, 19). The coordination exercise in these studies was conducted 10–45 min or 10 times/at least 3 times/day for 1–3 months. In contrast, stretch exercise, performed with or without coordination exercise (16, 18), did not show a significant effect on pain reduction in painful TMD patients. When pooling data on pain intensity on a 0–10 NRS for these 4 studies, these modalities of exercise therapy worked effectively to reduce pain intensity in the masticatory muscles (Standardized mean difference (SMD) −0.70, 95% confidence interval (CI) −0.99 to −0.42; 5 trials, 209 participants) (Figure 3A). The certainty of the evidence was judged to be low for this outcome (downgraded for bias and imprecision) (Figure 4).

When the intervention group and the control group were compared, 2 studies where coordination exercises were performed showed a significant effect on improving the ROM of the mandible (16, 19). In both studies, coordination exercise was conducted 10 min or 20 times/at least 3 times/day for 3–4 months. Pooled data on maximal mouth opening for these 2 studies showed that coordination exercise improved ROM in the mandible [Standardized mean difference (SMD) 0.36, 95% confidence interval (CI) 0.03–0.69; 4 trials, 145 participants] (Figure 3B). The certainty of the evidence of ROM was also judged to be low (downgraded for bias and imprecision) (Figure 4).

## 4. Discussion

This systematic review aimed to investigate the effect of exercise therapy on pain relief and improvement of TMJ mobility in patients with painful TMD conditions. The results indicated that coordination exercise can be most effective for TMD myalgia and arthralgia. In addition, stretch, excursion exercises, and resistance exercises also showed a positive effect on pain relief and TMJ mobility, however, there was no significant difference, compared to controls.

Stretching and coordination exercises, targeting specific mechanisms to improve jaw function and alleviate symptoms have been considered valuable approaches for TMD management. These exercises work by addressing muscle imbalances, enhancing jaw mobility, improving coordination, and promoting overall jaw health (21–23). The increased flexibility and extensibility of the muscles, ligaments, and connective tissues surrounding TMJ allow smoother and more comfortable jaw movements (24, 25). TMDs can disrupt the synchrony between the masticatory muscles in chewing and jaw function, leading to imbalances and disharmonized movement patterns. Through targeted coordination exercises that comprise mouth-opening and mouth-closing isotonic exercises, individuals can retrain their muscles to work together harmoniously, optimizing jaw function and reducing muscle imbalances (26, 27). Furthermore, stretching and coordination exercises contribute to proprioceptive awareness, allowing for better control and coordination of jaw movements. This, in turn, promotes a more stable and aligned TMJ. Abnormal muscle activation patterns and difficulties in controlling the jaw muscles are common in TMDs. By engaging in these exercises, individuals can rehabilitate and re-educate their muscles, promoting proper muscle activation, timing, and coordination. This rehabilitation process contributes to improved jaw function and reduced TMD symptoms (28). Finally, stretching and coordination exercises offer relaxation and stress reduction benefits. The focused and controlled movements involved in these exercises can help individuals experience a sense of calmness and relaxation. This can positively impact their overall well-being and help alleviate the stress associated with TMDs (29).

The findings in this study suggest that among the exercise modalities widely used for TMD management, coordination exercises demonstrate the highest potential to relieve pain and improve the mobility of the mandible. Different from stretching, coordination exercises involve active voluntary motion. For joint mobility, active exercise has been reported to be significantly more effective than passive exercise for patients with chronic low back pain (30). Moreover, the relationship between active voluntary movements with cognition and pain reduction has been reported in patients with non-specific low back pain (31). Another RCT showed a more robust effect of skilled training that requires motor cortex excitability on the improvement of motor coordination in low back pain (32). Our findings in this systematic review are aligned with and also could be explained by such change in brain plasticity caused by active voluntary exercise that requires motor control skills (33).

Our original protocol registered in PROSPERO includes both RCTs and observational studies as types of study to be included. However, throughout the process of this systematic review, we decided to focus on the results in the selected RCTs, considering the quality of scientific evidence. It was also because we would like to distinguish our review from the past reviews by establishing a clear definition of “P” and “I” in PICO. Furthermore, we aimed to obtain evidence that is easy for readers to understand by omitting confounding factors including study design in this case. On the other hand, as the identified papers were screened with the PICOS strictly set in this systematic review, a small number of studies were included. Therefore, the authors were not allowed to conduct a meta-analysis. This can be considered a limitation of this systematic review in terms of the review process. However, even though the heterogeneity of the selected studies was still detected, this review could identify the evidence narrowing down from our previous review (7). In addition, the small sample size also resulted in the low certainty of the evidence for pain intensity and ROM being downgraded due to serious study limitations and imprecision. Another point to be aware of is that the findings of this systematic review are based on the selected paper written in English, which could cause a bias for this systematic review. Also, the findings cannot be applied to specific age groups such as children under 12 years old or the elderly population, even though the population of the patients in the included papers covered the age range with a high prevalence of TMD (34).

Throughout the process of this systematic review, several concerns for further development of exercise therapy for TMDs were identified. First, terminology related to exercise therapy that has been used among dentists is not consistent with that in the medical field. Physical therapy, including exercise therapy, is one of the conservative treatment options for TMDs (35). Involvement of physiotherapists in TMD management is considered to be an immense advantage because physiotherapists could take a role to decrease pain intensity and restore normal masticatory function such as normal TMJ mobility in the treatment process of TMDs (36). However, the inconsistency in the terminology between the dental and medical fields could be a serious barrier that obstructs the propulsion of such a transdisciplinary approach to TMD management. Therefore, organizing definitions of the terminology used for exercise therapy would be essential as the first step.

In addition, the basic concept of the treatment structure of physiotherapy also needs to be aligned with the medical field. In the case of the situation in Japan, it appears that the classification of exercise modalities recommended for painful TMDs needs to be revisited. This is because the current classification of exercise modalities commonly used among dentists is not consistent with the one recognized among other medical-related occupations such as medical doctors, nurses, and physiotherapists. For example, manipulation that is classified as nonexercise therapy (37) in the medical field has been considered as one of the modalities categorized in exercise therapy in dentistry. The basic flow of physiotherapy treatment recommended by physiotherapists consists of 2 phases and each phase is divided into 3 steps (38). In Phase 1, therapeutical interventions aiming to obtain better physical conditions for exercise are conducted with modalities, which are applied also in dentistry, in the following manner; Step 1: Electrophysical agents (EPA) such as heat pack and Transcutaneous electrical nerve stimulation (TENS), Step 2: Manual therapy such as joint manipulation, Step 3: Local exercise therapy such as stretching, resistance training. Once local conditions such as joint mobility and muscle stretchability are prepared after Phase 1, instructive interventions in Phase 2 to improve or maintain the applicability of motion by active exercise learning and endurance training are to be conducted. As exercise therapy for TMDs mostly focuses on the masticatory muscles and the temporomandibular joint, the basic flow should be modified for patients with orofacial pain, and functional exercise therapy such as range of motion training, muscle endurance training, and coordination exercise can be included for dentists in Phase 2. An RCT showed a significant effect of active exercise therapy, including stretching and coordination exercises, performed after manual therapy on pain relief, as well as functional physical ability, in patients with chronic nonspecific low back pain, compared to exercise therapy only (39). Considering this evidence, there is a possibility that the confusion observed in dentistry mentioned above could mislead dentists to make an ineffective treatment plan and potentially result in unsatisfying outcomes for TMD patients, which should definitely be avoided. Therefore, there is a need to reconsider the classification as therapeutic modalities with physiotherapy first and to create clinical recommendations on how to proceed the physiotherapy including exercise therapy for patients with TMDs as an overall framework of treatment or management of TMDs.

Standardized clinical guidelines on physiotherapy for hip/knee osteoarthritis, shoulder pain, rheumatoid arthritis, and neck and low back pain have been established (37, 40–42). As a future goal, the launch of such guidelines for exercise therapy for TMDs should be prioritized. The FITT principle is widely recognized as a method of straightforward exercise prescription in the medical field (43–46). FITT stands for Frequency, Intensity, Time, and Type of therapeutic exercise and medical professionals can prescribe a concrete exercise program suitable for each patient according to target disorders or diseases. Based on the findings in this systematic review, the very first clue to establish FITT for painful TMD conditions was detected. In the near future, multi-institutional collaborative research needs to be conducted to collect clinical data sufficient for forming promising evidence and further define the optimal FITT for TMDs, as well as other orofacial pain conditions.

In conclusion, exercise therapy can be an effective option for managing orofacial pain and temporomandibular disorders (TMDs). However, due to some limitations such as low certainty of evidence, the effectiveness of exercise therapy for TMD management should still be investigated further. To improve the effectiveness of exercise therapy for TMDs, a revision of the classification of therapeutic modalities and terminology is essential for transdisciplinary collaboration. The establishment of standardized clinical guidelines and the application of the FITT principle for exercise prescription would also be beneficial for the future of exercise therapy for TMDs and other orofacial pain conditions. Further research is needed to collect sufficient clinical data to establish the optimal FITT for TMDs.

## Data Availability

The original contributions presented in the study are included in the article/**Supplementary Material**, further inquiries can be directed to the corresponding author.

## References

[1] Dworkin SF. Perspectives on the interaction of biological, psychological and social factors in TMD. J Am Dent Assoc. (1994) 125(7):856–63. 10.14219/jada.archive.1994.0212 8040536

[2] Kapos FP Exposto FG Oyarzo JF Durham J. Temporomandibular disorders: a review of current concepts in aetiology, diagnosis and management. Oral Surg. (2020) 13(4):321–34. 10.1111/ors.12473 34853604 PMC8631581

[3] List T Jensen RH. Temporomandibular disorders: old ideas and new concepts. Cephalalgia. (2017) 37(7):692–704. 10.1177/0333102416686302 28068790

[4] Schiffman E Ohrbach R Truelove E Look J Anderson G Goulet J Diagnostic criteria for temporomandibular disorders (DC/TMD) for clinical and research applications: recommendations of the international RDC/TMD consortium network* and orofacial pain special interest group†. J Oral Facial Pain Headache. (2014) 28(1):6–27. 10.11607/jop.1151 24482784 PMC4478082

[5] Batıbay S Külcü DG Kaleoğlu Ö Mesci N. Effect of pilates mat exercise and home exercise programs on pain, functional level, and core muscle thickness in women with chronic low back pain. J Orthop Sci. (2021) 26(6):979–85. 10.1016/j.jos.2020.10.026 33386201

[6] Nejati P Safarcherati A Karimi F. Effectiveness of exercise therapy and manipulation on sacroiliac joint dysfunction: a randomized controlled trial. Pain Physician. (2019) 22(1):53–61. 10.36076/ppj/2019.22.53 30700068

[7] Shimada A Ishigaki S Matsuka Y Komiyama O Torisu T Oono Y Effects of exercise therapy on painful temporomandibular disorders. J Oral Rehabil. (2019) 46(5):475–81. 10.1111/joor.12770 30664815

[8] Page MJ McKenzie JE Bossuyt PM Boutron I Hoffmann TC Mulrow CD The PRISMA 2020 statement: an updated guideline for reporting systematic reviews. Br Med J. (2021) 372:n71. 10.1136/bmj.n71 33782057 PMC8005924

[9] Page MJ Moher D Bossuyt PM Boutron I Hoffmann TC Mulrow CD PRISMA 2020 explanation and elaboration: updated guidance and exemplars for reporting systematic reviews. Br Med J. (2021) 372:n160. 10.1136/bmj.n160 33781993 PMC8005925

[10] Sasaki K Okeson JP, editors. Exercise therapy. In: The Japanese Society of Orofacial Pain, editors. The guide book of orofacial pain for diagnosis and treatment. 2nd ed. Tokyo: Ishiyaku Publishers, Inc. (2016). p. 136–40.

[11] Campbell M McKenzie JE Sowden A Katikireddi SV Brennan SE Ellis S Synthesis without meta-analysis (SWiM) in systematic reviews: reporting guideline. Br Med J. (2020) 368:l6890. 10.1136/bmj.l6890 31948937 PMC7190266

[12] Sterne JAC Savović J Page MJ Elbers RG Blencowe NS Boutron I Rob 2: a revised tool for assessing risk of bias in randomised trials. Br Med J. (2019) 366:l4898. 10.1136/bmj.l4898 31462531

[13] Guyatt GH Oxman AD Kunz R Vist GE Falck-Ytter Y Schünemann HJ. What is “quality of evidence” and why is it important to clinicians? Br Med J. (2008) 336(7651):995–8. 10.1136/bmj.39490.551019.BE 18456631 PMC2364804

[14] Guyatt GH Oxman AD Vist GE Kunz R Falck-Ytter Y Alonso-Coello P GRADE: an emerging consensus on rating quality of evidence and strength of recommendations. Br Med J. (2008) 336(7650):924–6. 10.1136/bmj.39489.470347.AD 18436948 PMC2335261

[15] Schünemann HJ Higgins JPT Vist GE Glasziou P Akl EA Skoetz N Chapter 14: completing summary of findings' tables and grading the certainty of the evidence. In: Higgins JPT Thomas J Chandler J Cumpston M Li T Page MJ Welch VA, editors. Cochrane handbook for systematic reviews of interventions version 6.3. Cochrane. (2022). Available at: www.training.cochrane.org/handbook

[16] Michelotti A Steenks MH Farella M Parisini F Cimino R Martina R. The additional value of a home physical therapy regimen versus patient education only for the treatment of myofascial pain of the jaw muscles: short-term results of a randomized clinical trial. J Orofac Pain. (2004) 18(2):114–25. 15250431

[17] de Felício CM de Oliveira MM da Silva MAMR. Effects of orofacial myofunctional therapy on temporomandibular disorders. Cranio. (2010) 28(4):249–59. 10.1179/crn.2010.033 21032979

[18] Craane B Dijkstra PU Stappaerts K De Laat A. One-year evaluation of the effect of physical therapy for masticatory muscle pain: a randomized controlled trial. Eur J Pain. (2012) 16(5):737–47. 10.1002/j.1532-2149.2011.00038.x 22337211

[19] Bae Y Park Y. The effect of relaxation exercises for the masticator muscles on temporomandibular joint dysfunction (TMD). J Phys Ther Sci. (2013) 25(5):583–6. 10.1589/jpts.25.583 24259807 PMC3804991

[20] Lindfors E Magnusson T Ernberg M. Effect of therapeutic jaw exercises in the treatment of masticatory myofascial pain: a randomized controlled study. J Oral Facial Pain Headache. (2020) 34(4):364–73. 10.11607/ofph.2670 33290442

[21] Michelotti A de Wijer A Steenks M Farella M. Home-exercise regimes for the management of non-specific temporomandibular disorders. J Oral Rehabil. (2005) 32(11):779–85. 10.1111/j.1365-2842.2005.01513.x 16202040

[22] Carlson CR Bertrand PM Ehrlich AD Maxwell AW Burton RG. Physical self-regulation training for the management of temporomandibular disorders. J Orofac Pain. (2001) 15(1):47–55. 11889647

[23] Moraes Ada R Sanches ML Ribeiro EC Guimarães AS. Therapeutic exercises for the control of temporomandibular disorders. Dental Press J Orthod. (2013) 18(5):134–9. 10.1590/s2176-94512013000500022 24352400

[24] Ucar M Sarp Ü Koca İ Eroğlu S Yetisgin A Tutoglu A Effectiveness of a home exercise program in combination with ultrasound therapy for temporomandibular joint disorders. J Phys Ther Sci. (2014) 26(12):1847–9. 10.1589/jpts.26.1847 25540479 PMC4273039

[25] Lee I-S Kim S-Y. Effectiveness of manual therapy and cervical spine stretching exercises on pain and disability in myofascial temporomandibular disorders accompanied by headaches: a single-center cohort study. BMC Sport Sci Med Rehabil. (2023) 15(1):39. 10.1186/s13102-023-00644-0 PMC10035158 36959659

[26] Marbach JJ. Temporomandibular pain and dysfunction syndrome. History, physical examination, and treatment. Rheum Dis Clin North Am. (1996) 22(3):477–98. 10.1016/s0889-857x(05)70283-0 8844909

[27] Nicolakis P Erdogmus B Kopf A Nicolakis M Piehslinger E Fialka-Moser V. Effectiveness of exercise therapy in patients with myofascial pain dysfunction syndrome. J Oral Rehabil. (2002) 29(4):362–8. 10.1046/j.1365-2842.2002.00859.x 11966970

[28] Liu S Fan S Li G Cai B Yao Y Jin L Short term effects of a novel combined approach compared with physical therapy alone among older patients with temporomandibular degenerative joint disease: a prospective cohort study. BMC Oral Health. (2023) 23(1):173. 10.1186/s12903-023-02848-9 36966303 PMC10040115

[29] Gil-Martínez A Paris-Alemany A López-de-Uralde-Villanueva I La Touche R. Management of pain in patients with temporomandibular disorder (TMD): challenges and solutions. J Pain Res. (2018) 11:571–87. 10.2147/JPR.S127950 29588615 PMC5859913

[30] Timm KE. A randomized-control study of active and passive treatments for chronic low back pain following L5 laminectomy. J Orthop Sports Phys Ther. (1994) 20(6):276–86. 10.2519/jospt.1994.20.6.276 7849747

[31] Wälti P Kool J Luomajoki H. Short-term effect on pain and function of neurophysiological education and sensorimotor retraining compared to usual physiotherapy in patients with chronic or recurrent non-specific low back pain, a pilot randomized controlled trial. BMC Musculoskelet Disord. (2015) 16:83. 10.1186/s12891-015-0533-2 25887550 PMC4413527

[32] Tsao H Galea MP Hodges PW. Driving plasticity in the motor cortex in recurrent low back pain. Eur J Pain. (2010) 14(8):832–9. 10.1016/j.ejpain.2010.01.001 20181504

[33] May A. Experience-dependent structural plasticity in the adult human brain. Trends Cogn Sci. (2011) 15(10):475–82. 10.1016/j.tics.2011.08.002 21906988

[34] Maixner W Fillingim RB Williams DA Smith SB Slade GD. Overlapping chronic pain conditions: implications for diagnosis and classification. J Pain. (2016) 17(9 Suppl):T93–T107. 10.1016/j.jpain.2016.06.002 27586833 PMC6193199

[35] Romero-Reyes M Uyanik JM. Orofacial pain management: current perspectives. J Pain Res. (2014) 7:99–115. 10.2147/JPR.S37593 24591846 PMC3937250

[36] Herrero Babiloni A Lam JTAT Exposto FG Beetz G Provost C Gagnon DH Interprofessional collaboration in dentistry: role of physiotherapists to improve care and outcomes for chronic pain conditions and sleep disorders. J Oral Pathol Med. (2020) 49(6):529–37. 10.1111/jop.13068 32531851

[37] van Doormaal MCM Meerhoff GA Vliet Vlieland TPM Peter WF. A clinical practice guideline for physical therapy in patients with hip or knee osteoarthritis. Musculoskeletal Care. (2020) 18(4):575–95. 10.1002/msc.1492 32643252

[38] Kimura T. The concept of physiotherapy. In: Kimura T Takahashi T Uchi M, editors. Foundations of therapeutic exercise and clinical practice. 1st ed. Tokyo: KANEHARA & Co., Ltd. (2020). p. 14–22.

[39] Balthazard P de Goumoens P Rivier G Demeulenaere P Ballabeni P Dériaz O. Manual therapy followed by specific active exercises versus a placebo followed by specific active exercises on the improvement of functional disability in patients with chronic non specific low back pain: a randomized controlled trial. BMC Musculoskelet Disord. (2012) 13:162. 10.1186/1471-2474-13-162 22925609 PMC3518179

[40] Klintberg IH Cools AMJ Holmgren TM Holzhausen AG Johansson K Maenhout AG Consensus for physiotherapy for shoulder pain. Int Orthop. (2015) 39(4):715–20. 10.1007/s00264-014-2639-9 25548127

[41] Peter WF Swart NM Meerhoff GA Vliet Vlieland TPM. Clinical practice guideline for physical therapist management of people with rheumatoid arthritis. Phys Ther. (2021) 101(8):1–16. 10.1093/ptj/pzab127 34003240

[42] Corp N Mansell G Stynes S Wynne-Jones G Morsø L Hill JC Evidence-based treatment recommendations for neck and low back pain across Europe: a systematic review of guidelines. Eur J Pain. (2021) 25(2):275–95. 10.1002/ejp.1679 33064878 PMC7839780

[43] ACSM. ACSM's guidelines for exercise testing and prescription. 9th ed. (Lupash E, ed.). Philadelphia: Lippincott Williams & Wilkins (2014) p. 91–114.

[44] Billinger SA Boyne P Coughenour E Dunning K Mattlage A. Does aerobic exercise and the FITT principle fit into stroke recovery? Curr Neurol Neurosci Rep. (2015) 15(2):519. 10.1007/s11910-014-0519-8 25475494 PMC4560458

[45] Pescatello LS Franklin BA Fagard R Farquhar WB Kelley GA Ray CA. American college of sports medicine position stand. Exercise and hypertension. Med Sci Sports Exerc. (2004) 36(3):533–53. 10.1249/01.mss.0000115224.88514.3a 15076798

[46] O’Riordan C Clifford A Van De Ven P Nelson J. Chronic neck pain and exercise interventions: frequency, intensity, time, and type principle. Arch Phys Med Rehabil. (2014) 95(4):770–83. 10.1016/j.apmr.2013.11.015 24333741

