# Supplementary material for: Effectiveness of exercise therapy on pain relief and jaw mobility in patients with pain-related temporomandibular disorders: a systematic review
Source: Front Oral Health. 2023 Jul 12;4:1170966. doi: 10.3389/froh.2023.1170966 (PMC10382173; doi:10.3389/froh.2023.1170966)
Supplement: Supplementary file 1 [file Table1.docx]

| Search strategy and Algorisms for PubMed | | |
| --- | --- | --- |
| #1 | Population | ((Orofacial[All Fields] OR ("face"[MeSH Terms] OR "face"[All Fields] OR "facial"[All Fields]) OR ("face"[MeSH Terms] OR "face"[All Fields])) AND ("pain"[MeSH Terms] OR "pain"[All Fields])) |
| #2 | Intervention | (("exercise"[MeSH Terms] OR "exercise"[All Fields]) OR ("physical therapy modalities"[MeSH Terms] OR ("physical"[All Fields] AND "therapy"[All Fields] AND "modalities"[All Fields]) OR "physical therapy modalities"[All Fields] OR ("physical"[All Fields] AND "therapy"[All Fields]) OR "physical therapy"[All Fields]) OR ("physical therapy modalities"[MeSH Terms] OR ("physical"[All Fields] AND "therapy"[All Fields] AND "modalities"[All Fields]) OR "physical therapy modalities"[All Fields] OR "physiotherapy"[All Fields])) |
| #3 | Study design | (("randomized controlled trial"[Publication Type] OR "randomized controlled trials as topic"[MeSH Terms] OR "randomized controlled trial"[All Fields] OR "randomized controlled trial"[All Fields]) OR ("controlled clinical trial"[Publication Type] OR "controlled clinical trials as topic"[MeSH Terms] OR "controlled clinical trial"[All Fields]) OR ("random allocation"[MeSH Terms] OR ("random"[All Fields] AND "allocation"[All Fields]) OR "random allocation"[All Fields]) OR ("double-blind method"[MeSH Terms] OR ("double-blind"[All Fields] AND "method"[All Fields]) OR "double-blind method"[All Fields] OR ("double"[All Fields] AND "blind"[All Fields] AND "method"[All Fields]) OR "double blind method"[All Fields]) OR ("single-blind method"[MeSH Terms] OR ("single-blind"[All Fields] AND "method"[All Fields]) OR "single-blind method"[All Fields] OR ("single"[All Fields] AND "blind"[All Fields] AND "method"[All Fields]) OR "single blind method"[All Fields]) OR ("clinical trial"[Publication Type] OR "clinical trials as topic"[MeSH Terms] OR "clinical trial"[All Fields]) OR ("placebos"[MeSH Terms] OR "placebos"[All Fields] OR "placebo"[All Fields]) OR ("random allocation"[MeSH Terms] OR ("random"[All Fields] AND "allocation"[All Fields]) OR "random allocation"[All Fields] OR "random"[All Fields]) OR ("evaluation studies"[Publication Type] OR "evaluation studies as topic"[MeSH Terms] OR "evaluation studies"[All Fields]) OR ("follow-up studies"[MeSH Terms] OR ("follow-up"[All Fields] AND "studies"[All Fields]) OR "follow-up studies"[All Fields] OR ("follow"[All Fields] AND "up"[All Fields] AND "studies"[All Fields]) OR "follow up studies"[All Fields]) OR ("prospective studies"[MeSH Terms] OR ("prospective"[All Fields] AND "studies"[All Fields]) OR "prospective studies"[All Fields]) OR ("cross-over studies"[MeSH Terms] OR ("cross-over"[All Fields] AND "studies"[All Fields]) OR "cross-over studies"[All Fields] OR ("cross"[All Fields] AND "over"[All Fields] AND "studies"[All Fields]) OR "cross over studies"[All Fields]) OR ("prevention and control"[Subheading] OR ("prevention"[All Fields] AND "control"[All Fields]) OR "prevention and control"[All Fields] OR "control"[All Fields] OR "control groups"[MeSH Terms] OR ("control"[All Fields] AND "groups"[All Fields]) OR "control groups"[All Fields]) OR ("longitudinal studies"[MeSH Terms] OR ("longitudinal"[All Fields] AND "studies"[All Fields]) OR "longitudinal studies"[All Fields] OR "prospective"[All Fields])) |
| Final search |  | #1 AND #2 AND #3 |
